# The effect of virtual reality on anxiety and pain in patients undergoing totally implantable venous access port: a systematic review and meta-analysis

**DOI:** 10.3389/fmed.2026.1923678

**Published:** 2026-09-08

**Authors:** Qingqing Zhu, Wei Xie, Yuanqian Luo, Ping Yu, Zehua Shu, Gaolei Liu

**Affiliations:** 1 Department of Urology, Daping Hospital, Army Medical University, Chongqing, China; 2 Department of Anesthesiology, Daping Hospital, Army Medical University, Chongqing, China

**Keywords:** anxiety, meta-analysis, pain, systematic review, totally implantable venous access port, virtual reality

## Abstract

**Background:**

The totally implantable venous access port (TIVAP) is the preferred venous access device for patients requiring long-term chemotherapy or intravenous nutrition. However, as an invasive procedure, TIVAP implantation often induces considerable anxiety and pain. Virtual reality (VR), a non-pharmacological distraction technique, provides an immersive audio-visual experience that redirects patients' attention away from noxious stimuli, and has been proven to effectively alleviate pain and anxiety across various medical procedures. Randomized controlled trials (RCTs) have reported that VR interventions significantly reduce pain and anxiety levels during TIVAP implantation. Nevertheless, a comprehensive synthesis of evidence on the effectiveness of VR intervention in this specific setting is currently lacking. Therefore, this study aimed to evaluate, through a meta-analysis, the effects of VR intervention on anxiety and pain in patients undergoing TIVAP implantation, with the goal of providing evidence-based guidance for clinical practice.

**Methods:**

We systematically searched PubMed, Cochrane Library, Web of Science, and Embase for RCTs examining the effects of VR intervention on anxiety and pain in patients receiving TIVAP implantation, from inception to June 2026. Two researchers independently screened the literature, extracted data, and assessed the methodological quality of included studies using the Cochrane Risk of Bias 2.0 (RoB2) tool. The primary outcomes were anxiety and pain scores. A meta-analysis was conducted using RevMan 5.3 software, and the quality of evidence for each outcome was rated using the Grading of Recommendations Assessment, Development and Evaluation (GRADE) system.

**Results:**

Five studies involving 452 participants were included. The meta-analysis revealed no no statistically significant difference between VR group and the control group in improving pain scores [SMD = −0.84, 95% CI (−1.89, 0.22), *P* = 0.119; Evidence level: Very low]. However, VR intervention was associated with a statistically significant reduction in anxiety scores, albeit with low-quality evidence and uncertain clinical significance [SMD = −1.89, 95% CI (−3.42, −0.36), *P* = 0.015; Evidence level: Low]. Subgroup analyses indicated that, for pain scores, studies with a sample size of <100 showed a significant pain-reducing effect of VR intervention, whereas those with ≥100 participants did not. When stratified by control group type, VR intervention did not significantly reduce pain scores compared with either standard care or music intervention. For anxiety scores, VR intervention significantly alleviated anxiety in both sample-size subgroups (<100 and ≥100). Stratified by control type, VR intervention demonstrated a significant anxiety-reducing effect when the control group received music intervention, but the difference did not reach statistical significance when compared with standard care.

**Conclusions:**

Current evidence is insufficient to support a definitive clinical benefit of VR intervention in alleviating anxiety and pain among patients undergoing TIVAP implantation. Although VR intervention offers theoretical advantages as a non-pharmacological approach, its efficacy appears to be influenced by multiple factors, including device characteristics, content, duration, and application context. Given the low strength of available evidence, routine adoption of VR intervention as an adjunctive measure cannot be strongly recommended at this time. Future research should involve larger sample sizes, rigorous designs, standardized VR intervention protocols, and unified outcome assessment tools to provide more robust evidence for clinical decision-making.

**Systematic Review Registration:**

https://www.crd.york.ac.uk/PROSPERO/view/CRD420261430609, identifier CRD420261430609.

## Introduction

The totally implantable venous access port (TIVAP) is the preferred central venous access device for patients undergoing long-term chemotherapy or parenteral nutrition support (1, 2). It offers significant advantages, including a low infection rate, ease of maintenance, and minimal interference with daily life (3, 4). However, TIVAP implantation is an invasive procedure; although performed under local anesthesia, most patients experience varying degrees of anxiety and pain before, during, and after the procedure. Current evidence indicates that these anxiety and pain symptoms not only compromise patients' psychological well-being and treatment adherence but may also induce hemodynamic instability, elevate surgical risks, and even foster long-term fear and avoidance of medical procedures (5, 6).

Routine clinical interventions mainly encompass preoperative verbal reassurance, adequate local anesthesia, and oral anxiolytics (7, 8). However, pharmacological approaches are limited by variable onset, inter-individual response differences, and potential side effects. Non-pharmacological interventions, meanwhile, lack standardized protocols and have shown suboptimal overall efficacy. Therefore, it is urgent to find a safe, effective, and easily scalable non-drug auxiliary intervention measure to improve the perioperative experience of patients with TIVAP implantation.

Virtual reality (VR) intervention is an emerging digital distraction tool (9, 10) that delivers a realistic three-dimensional audiovisual environment via a head-mounted display, effectively blocking or attenuating external noxious stimuli (11, 12). VR intervention has been shown to alleviate pain and reduce anxiety across diverse settings, including burn wound care, dental procedures, invasive interventions, and postoperative rehabilitation (13–16). The therapeutic effects of VR intervention on pain and anxiety are underpinned by multiple interrelated mechanisms. At the cognitive level, attention distraction theory posits that immersive VR experiences compete for limited attentional resources within the brain's central executive system, thereby reducing the capacity to process nociceptive and threat-related signals (17, 18). However, growing evidence suggests that the analgesic and anxiolytic effects of VR extend beyond simple distraction (19–21). From a neurobiological perspective, immersive VR experiences simultaneously engage visual, auditory, and somatosensory cortices; by inducing a state of sensory enrichment, they modulate pain perception through descending inhibitory pathways (22, 23). Functional neuroimaging studies have shown that VR intervention can reduce pain-related neural activity in the anterior cingulate cortex, insula, and primary somatosensory cortex, while activating prefrontal regions involved in cognitive reappraisal and emotional regulation (23, 24). In addition, VR intervention often incorporate relaxation-inducing elements, such as guided breathing, calm visual scenes, and hypnotic suggestions. These can directly influence sympathetic nervous system excitability and hypothalamic-pituitary-adrenal axis activity (25–27), as confirmed by reductions in heart rate, blood pressure, and salivary cortisol levels after VR exposure (28, 29). These physiological changes may be particularly relevant to anxiety reduction, as anxiety is closely linked to autonomic hyperarousal and the amygdala-centered fear neurocircuitry (30–32). Notably, the relative contributions of these mechanisms may vary depending on VR content, exposure duration, and clinical context.

Notably, TIVAP implantation presents specific characteristics that make VR particularly suitable. Although the procedure is minimally invasive, patients typically remain awake and can clearly perceive steps such as puncture, dilation, and catheter placement, and the stressful operating room environment often triggers marked anxiety and pain. Unlike surgeries under general anesthesia, the patient's full consciousness during local anesthesia gives psychological intervention unique value. The procedure usually lasts 30–60 min, a time window that is suitable for short-term VR immersion—long enough to cover the most intensely stimulating phases through distraction, yet not so long as to cause fatigue or discomfort. Most patients receiving TIVAP are those with malignant tumors undergoing long-term chemotherapy, who have often endured multiple venipunctures, infusions, and treatment-related traumatic experiences, leading to high anticipatory anxiety and cumulative fear of medical procedures. Such patients are more psychologically vulnerable to invasive interventions; simple preoperative verbal reassurance is often insufficient to reduce their anxiety, and more effective non-pharmacological adjuncts are urgently needed. During awake invasive procedures, VR occupies both visual and auditory channels, effectively blocking the transmission of external noxious stimuli and reducing the brain's allocation of processing resources to pain and fear signals. Thus, it provides a “psychological supplementary analgesia” on top of “pharmacological pain blockade,” creating a synergistic effect of drug and non-drug interventions.

In recent years, several randomized controlled trials (RCTs) have applied VR intervention during TIVAP implantation and reported encouraging initial findings. However, these studies vary considerably in sample size, VR equipment, intervention content and duration, and outcome assessment tools, leading to inconsistent conclusions. Moreover, the limited statistical power of individual studies precludes a definitive clinical recommendation. To date, no meta-analysis has systematically synthesized the evidence in this field. Such a synthesis is lacking, making it difficult for clinicians to obtain a quantitative summary of VR's effects in this specific context, thereby hindering evidence-based decision-making regarding the incorporation of VR into perioperative care for TIVAP patients. Therefore, this study aims to conduct a meta-analysis of RCTs evaluating the effects of VR intervention on anxiety and pain management in adult patients undergoing TIVAP implantation. The specific objectives are: (1) to quantitatively synthesize the pooled effects of VR intervention on anxiety and pain scores; (2) to explore potential sources of heterogeneity through subgroup analyses based on sample size and control group type; and (3) to assess the certainty of evidence using the GRADE framework. By systematically integrating the available evidence, this meta-analysis aims to provide more precise and reliable estimates of the effects of VR intervention in this population, identify evidence gaps worthy of further investigation, and offer clinicians an evidence-based reference when considering VR intervention as a non-pharmacological adjunct during TIVAP implantation. The findings are expected to clarify the current state of evidence, elucidate the conditions under which VR intervention may be most effective, and identify priorities for future high-quality research.

## Methods

This study was conducted in accordance with the updated Preferred Reporting Items for Systematic Reviews and Meta-Analyses (PRISMA) guidelines (33) and was registered in PROSPERO (CRD420261430609).

### Search strategy

We searched four major English-language databases—PubMed, Cochrane Library, Web of Science, and Embase—from their inception to June 2026, without language restrictions. Both published articles and preprints were considered eligible. The search strategy combined MeSH terms and free words, including “virtual reality”, “VR”, “totally implantable venous access port”, “TIVAP”, “anxiety”, “pain”, “randomized controlled trial”, etc., with appropriate adaptations for each database's syntax (Supplementary Materials Table S1. Search strategy). Additionally, we manually screened the reference lists of included studies to identify further potentially relevant literature.

### Inclusion and exclusion criteria

Studies were included if they met all the following criteria:

(1) P (Population): patients aged >18 years undergoing TIVAP implantation; (2) I (Intervention): the intervention group received VR intervention in addition to routine procedures, including but not limited to immersive audiovisual content delivered via head-mounted displays; no restrictions were placed on VR device type, content theme, duration, or timing of application; (3) C (Comparison): the control group received standard care (routine procedures) or music intervention; (4) O (Outcomes): primary outcomes were anxiety and pain scores, measured using any validated quantitative scale (e.g., NRS, VAS, STAI-6, SAI, McGill Pain Questionnaire); (5) S (Study design): RCTs, irrespective of blinding or publication language.

Studies were excluded if they met any of the following criteria:

(1) Case reports, reviews, conference abstracts, animal experiments, or basic research; (2) duplicate publications or studies with incomplete data; (3) full text unavailable or outcome data not reported; (4) intervention groups that received other active non-pharmacological interventions concurrently with VR intervention.

### Data extraction

Two researchers independently screened titles and abstracts for initial eligibility, then read full texts of potentially relevant or uncertain articles to make final decisions. Discrepancies were resolved through discussion or consultation with a third researcher. Reasons for exclusion were recorded, and a PRISMA flow diagram was generated. Data were extracted on: (1) basic study information (first author, year, country/region); (2) participant characteristics (sample size, age, sex, main tumor type); (3) VR intervention details (device type, content description, duration, audio usage); (4) control group intervention type; (5) outcome measures (names of anxiety and pain scales, with mean, standard deviation, and sample size per group); (6) methodological features (randomization method, allocation concealment, blinding implementation). Extraction was performed independently by two researchers and cross-checked; disagreements were settled by discussion.

### Risk of bias

The risk of bias of the included RCTs was assessed using the Cochrane Risk of Bias 2.0 (RoB2) tool. The RoB2 tool evaluates bias across five domains: (1) the randomization process (sequence generation and allocation concealment); (2) deviations from intended interventions (including blinding of participants and personnel); (3) missing outcome data; (4) measurement of the outcome (blinding of outcome assessors); and (5) selective reporting of results. Each domain is rated as “low risk”, “some concerns”, or “high risk”. An overall risk-of-bias judgment is then derived based on the combined ratings across the five domains. Two researchers independently assessed the risk of bias for all included studies, and the results were cross-checked. Disagreements were resolved through discussion and, if necessary, by consultation with a third researcher. Given the nature of VR interventions—participants cannot be blinded once wearing a head-mounted display, and clinicians performing the procedure cannot be blinded due to the obvious differences in intervention modes—this study followed the RoB2 guidance by rating the “deviations from intended interventions” domain as “high risk” for unblinding resulting from the intervention's nature. This judgment aligns with the RoB2 recommendations for non-pharmacological intervention trials.

### Statistical analysis

Meta-analyses were performed using RevMan 5.3 software. Because the assessment tools for anxiety and pain varied across studies, we used the standardized mean difference (SMD) with 95% confidence interval (CI) as the summary statistic to eliminate dimensional differences. Heterogeneity was assessed using the *χ*^2^ test and the *I*^2^ statistic. If *I*^2^ ≤ 50% and *P* ≥ 0.10, we considered the studies homogeneous and used a fixed-effect model (34); if *I*^2^ > 50% and *P* < 0.10, we considered heterogeneity significant and used a random-effects model, with cautious interpretation of results (35). For the random-effects model, between-study variance (*τ*^2^) was estimated using the DerSimonian-Laird (DL) method, which is the most commonly used approach in random-effects meta-analysis due to its computational simplicity and favorable statistical properties. Weights were assigned using the inverse-variance method (i.e., each study was weighted by the reciprocal of its variance). To explore sources of heterogeneity, we performed subgroup analyses based on: (1) sample size (<100 vs. ≥100), and (2) control group type (standard care vs. music intervention). Specifically, sample size was chosen to assess the potential impact of small-study effects on pooled estimates; smaller studies are more susceptible to random error, publication bias, and overestimation, whereas larger studies typically provide more stable and conservative estimates. In the field of VR intervention research, many early trials used limited samples, so it was necessary to evaluate whether the overall effect was driven mainly by small studies. The threshold of 100 was selected because it approximates the median sample size of the included studies and represents a practical minimum sample size for detecting a moderate effect size in a parallel-group RCT. Control group type was chosen as a subgroup variable because these two comparators represent fundamentally different baselines: standard care reflects a “no intervention” baseline, allowing assessment of VR's absolute effect, whereas music intervention serves as an active non-pharmacological control, enabling us to determine whether VR intervention offers additional benefit beyond a simple, low-cost auditory distraction. This distinction is clinically meaningful because if VR intervention does not outperform music, its added value in routine practice would be questionable, especially given its higher equipment costs and operational complexity. Sensitivity analysis was performed using sequential exclusion of individual studies. Because fewer than 10 studies were included, we did not conduct the Egger test but used funnel plot inspection for qualitative assessment of publication bias.

### Evaluation of evidence quality

The Grading of Recommendations Assessment, Development and Evaluation (GRADE) system was used to rate the certainty of evidence for the primary outcomes (anxiety and pain scores). Evidence quality was classified into four levels: high (⊕⊕⊕⊕), moderate (⊕⊕⊕○), low (⊕⊕○○), and very low (⊕○○○). Because all included studies were RCTs, the initial evidence quality was rated as high. We then downgraded for each of the five factors: (1) risk of bias; (2) inconsistency; (3) indirectness; (4) imprecision; (5) publication bias. Grading was performed independently by two researchers, with disagreements resolved through discussion.

## Results

### Searching results

**Figure 1:**
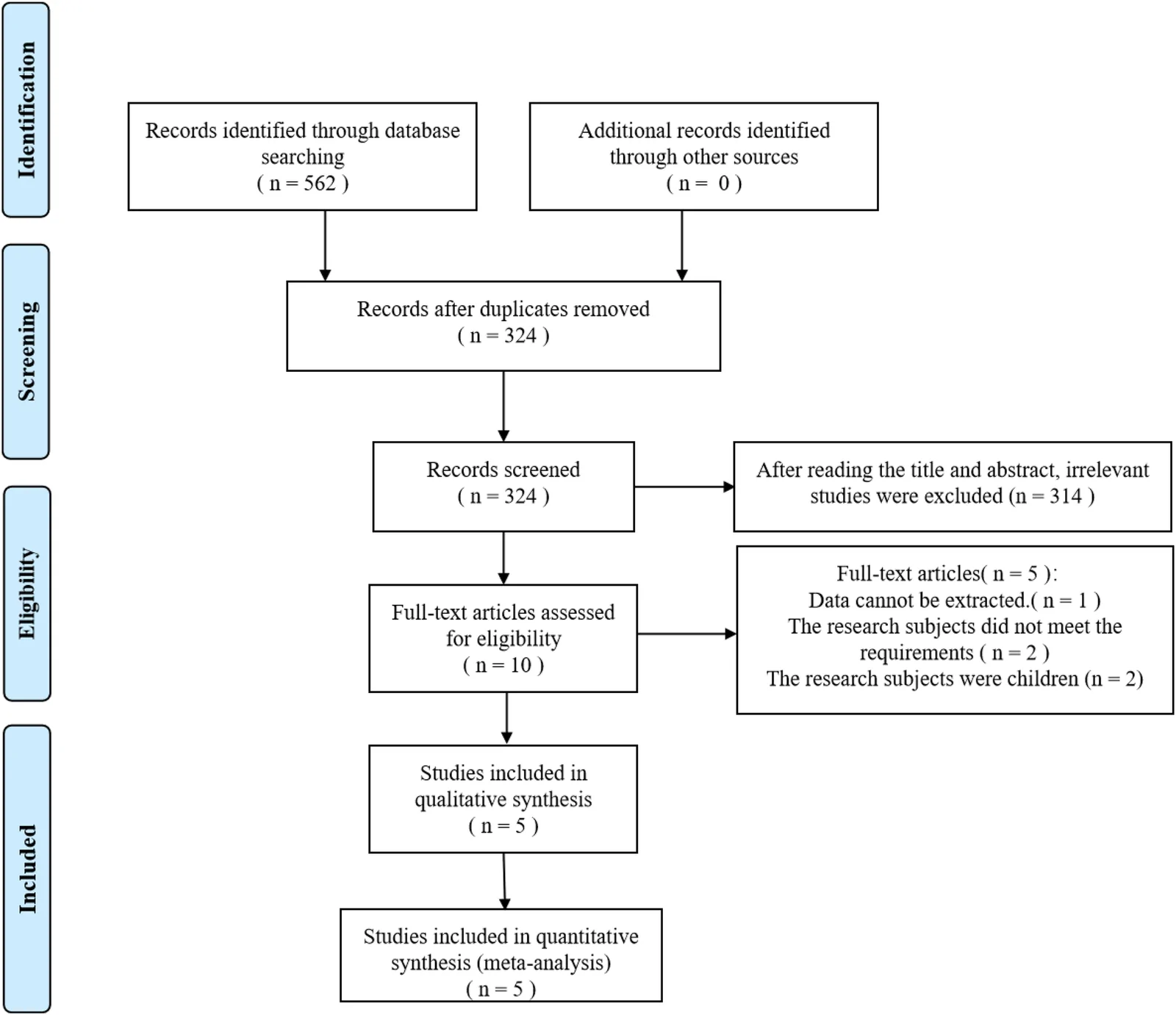
PRISMA 2020 literature screening flowchart. PRISMA, preferred reporting items for systematic reviews and meta-analyses.

A total of 562 records were initially identified. After removing duplicates using EndNote, 215 records remained. By reading the titles and abstracts, 238 studies were excluded because they were reviews, non-RCTs, case reports, or irrelevant to the topic. Consequently, 10 articles were retrieved for full-text assessment. Following full-text reading, 5 articles were excluded, and finally 5 RCTs (15, 36–39) were included in the meta-analysis (Figure 1).

### Characteristics of eligible studies

A total of 5 studies (15, 36–39) were included, with publication years ranging from 2022 to 2026. These studies were conducted in four countries: France, Japan, Turkey, and Germany, involving a total of 452 participants. The sample size per study ranged from 10 to 139 patients. The mean age of participants ranged from 42 to 63 years. The main types of tumors included breast cancer, colorectal cancer, lung cancer, and pancreatic cancer, etc. Regarding VR equipment, four studies (15, 36, 38, 39) used PICO G2 4K headsets, and one study (37) used smartphone-compatible VR glasses. The VR intervention content included underwater journeys, natural landscapes, space exploration, and hypnosis-guided imagery, etc. The duration of the VR intervention varied from 1 min before the surgery to 4 h post-operatively; two studies applied VR intervention throughout the entire surgical procedure. For outcome assessment, pain was measured using the NRS (3 studies) (15, 36, 39), VAS (1 study) (37), and the Short McGill Pain Questionnaire (1 study) (38). Anxiety was assessed using the NRS (1 study) (36), VAS (1 study) (15), SAI (1 study) (37), and STAI-6 (2 studies) (38, 39). The basic characteristics of the included studies are summarised in Table 1.

**Table 1:** The basic characteristics of the included trials.

Study	Region	Study period	Total sample size	Age (years)	Female	Main cancer types	Anxiety Assessment Tool	Pain Assessment Tool	Operator characteristics	Anesthesia method	Virtual reality intervention	Control group	Pain assessment time	Anxiety assessment time	Follow-up duration
Ghimouz et al. (36)	France	From May 2022 to August 2023	127	54 ± 12/57 ± 15	87%	Breast cancer (60%)	NRS	NRS	An experienced anesthesiologist	1% lidocaine 20 mL + sodium bicarbonate 4 mL	Equipment: PICO G2 4 K headset.	Standard Care Group (STAND): Received the standard CV port insertion procedure but did not receive VR intervention.	Within 15 min after the operation	Within 15 min after the operation	NR
											Content: “AQUA” underwater journey by OnComfort-Sedakit company (including self-hypnosis suggestions, adjusting breathing while following the whales).				
											Duration: 30–45 min (adjustable).				
											Audio: Headset built-in speakers (no headphones used to maintain communication).				
Kamada et al. (15)	Japan	From July 2024 to October 2024	10	63.1 ± 10.6	50%	Colorectal cancer (70%)	VAS	NRS	Senior resident physician	1% Secocaine 20 mL	Equipment: PICO G2 headset.	Standard Care Group (STAND): Received the standard CV port insertion procedure but did not receive VR intervention.	Immediately after the operation	During the operation and immediately after the operation	NR
											Content: Therapeia VR software (including space, forest, and ocean scenes, with voice-guided breathing rhythms to promote relaxation).				
											Duration: Used throughout the surgery.				
											Audio: Guiding breathing voice.				
Menekli et al. (37)	Turkey	From September 2019 to January 2020	139	42-52	58%	Breast cancer/Lung cancer	SAI	VAS	NR	2% lidocaine	Equipment: Turkcell TV-VR glasses (compatible with smartphones, 4.7–6 inches).	Standard Care Group (STAND): Received the standard CV port insertion procedure but did not receive VR intervention.	Before the operation, during the operation (at the 25th minute), and 4 h after the operation	Before the operation and after the operation	NR
											Content: Various 3–10 min videos (park, natural beach, underwater world, museum tour) + theme relaxation music, patients can choose themselves.				
											Duration: Begins 1 min before the surgery and lasts until 4 h after the surgery (as needed).				
											Audio: Integrated music from the video.				
Sargut et al. (38)	Germany	From February 22, 2022 to January 13, 2023	60	NR	43.3%/50%	NR	STAI-6	Short McGill Pain Questionnaire	NR	2% Procaine 20 mL	Equipment: Pico G2 4 K Premium headset (provided by SyncVR Medical).	Music Control Group: No VR headset was used, but participants could choose their own music programs to listen to.	After the operation	Before the operation and after the operation	NR
											Content: SyncVR Relax & Distract program [including underwater world, beach, winter landscape, forest walk, space, etc. scenes, and users can choose background music (jazz/light music/classical)].				
											Duration: Used throughout the surgery.				
											Audio: Built-in background music for the scenes.				
Steinkraus et al. (39)	Germany	From January 2022 to August 2023	116	62 ± 13.6/63 ± 12	41.7%/23.2%	Colorectal cancer (22.4%)/Pancreatic cancer (16.4%)	STAI-6	NRS	Surgical resident (supervised by senior physicians)	0.75% Ropivacaine 20 mL	Equipment: PICO G2 4 K headset.	Standard Care Group (STAND): Received the standard CV port insertion procedure but did not receive VR intervention.	Before the operation, immediately after the operation, and before discharge (approximately 2 h after the operation)	Before the operation, immediately after the operation, and before discharge (approximately 2 h after the operation)	30 days after the operation
											Content: HypnoVR CE certified software [including 6 environments: “Winter Magic, Forest, Tropical Beach, Deep Sea Diving, Space Voyage, Four Seasons”; optional music atmosphere (relaxation/serenity/wind band, etc.); optional male/female voice guidance audio].				
											Duration: Entire surgical procedure.				
											Audio: Headset built-in speakers (deliberately not wearing headphones to facilitate communication with medical staff during the procedure).				

NRS, Numeric Rating Scale; VAS, visual analogue scale; SAI, State anxiety; STAI-6, State-Trait Anxiety Inventory; NR, not report.

**Figure 2:**
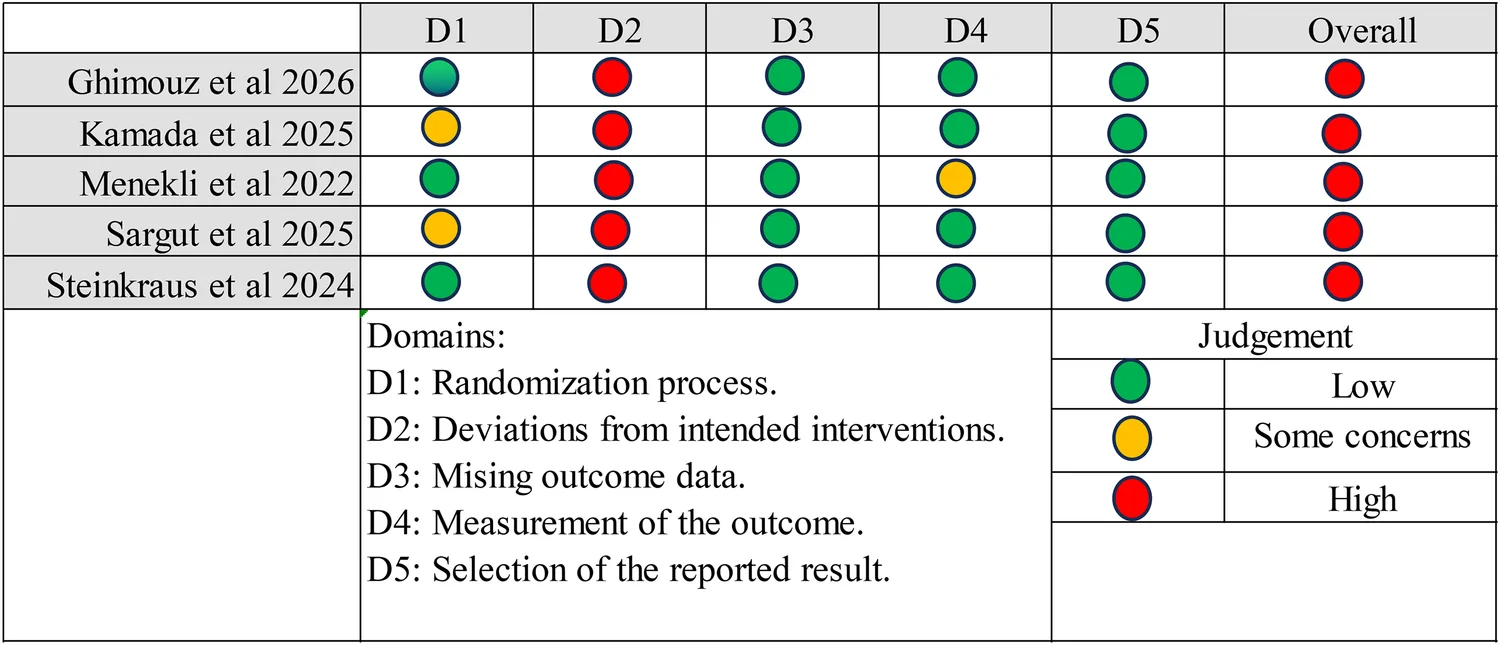
Summary chart for assessing the risk of bias in included studies.

All five studies (15, 36–39) were rated as having a high risk of bias in the “deviations from intended interventions” domain, because blinding of participants and personnel delivering the intervention was not feasible due to the inherent nature of VR, inevitably introducing substantial performance bias. Two studies (15, 38) had “some concerns” in the “randomisation process” domain, mainly due to inadequate reporting of randomisation methods and allocation concealment. One study (37) had “some concerns” in the “measurement of the outcome” domain, as it did not clearly specify whether outcome assessors were blinded. According to the RoB 2 criteria, because all studies had a high risk of bias arising from the inability to blind, the overall risk of bias was judged as high for all five studies. The results of the risk-of-bias assessment are presented in Figure 2.

### Meta-analysis

**Figure 3:**
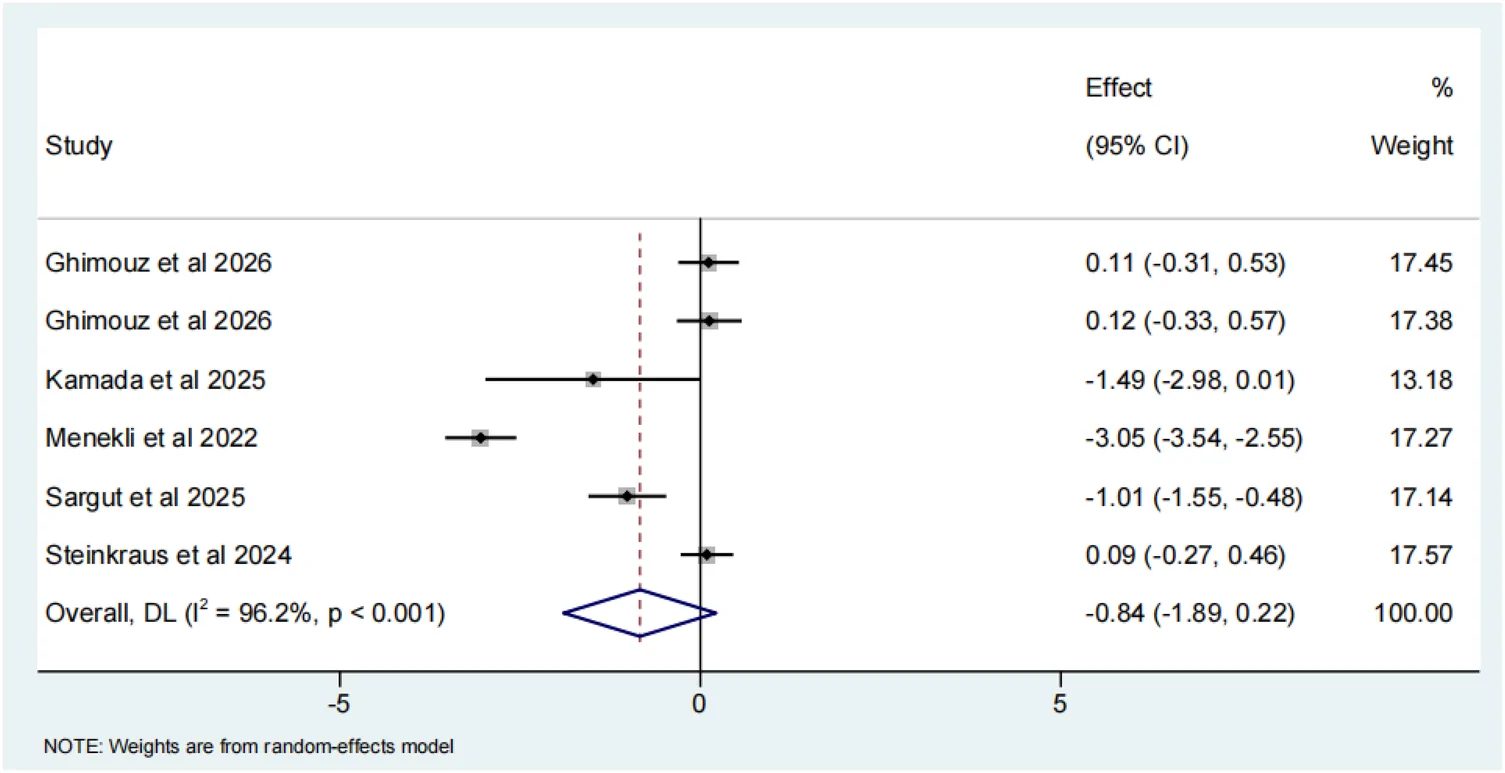
Forest plot showing the effect of VR intervention on pain scores of patients with TIVAP implantation. CI, confidence interval; DL, Random effects model; VR, virtual reality; TIVAP, totally implantable venous access port.

A total of 5 studies (15, 36–39) were included for pain scores. The heterogeneity test showed *I*^2^ = 96.20%, *P* < 0.001,prompting the use of a random-effects model. The meta-analysis revealed no statistically significant difference between the VR intervention group and the control group in improving the pain scores in patients undergoing TIVAP implantation [SMD = −0.84, 95% CI (−1.89, 0.22), *P* = 0.119] (Figure 3).

**Figure 4:**
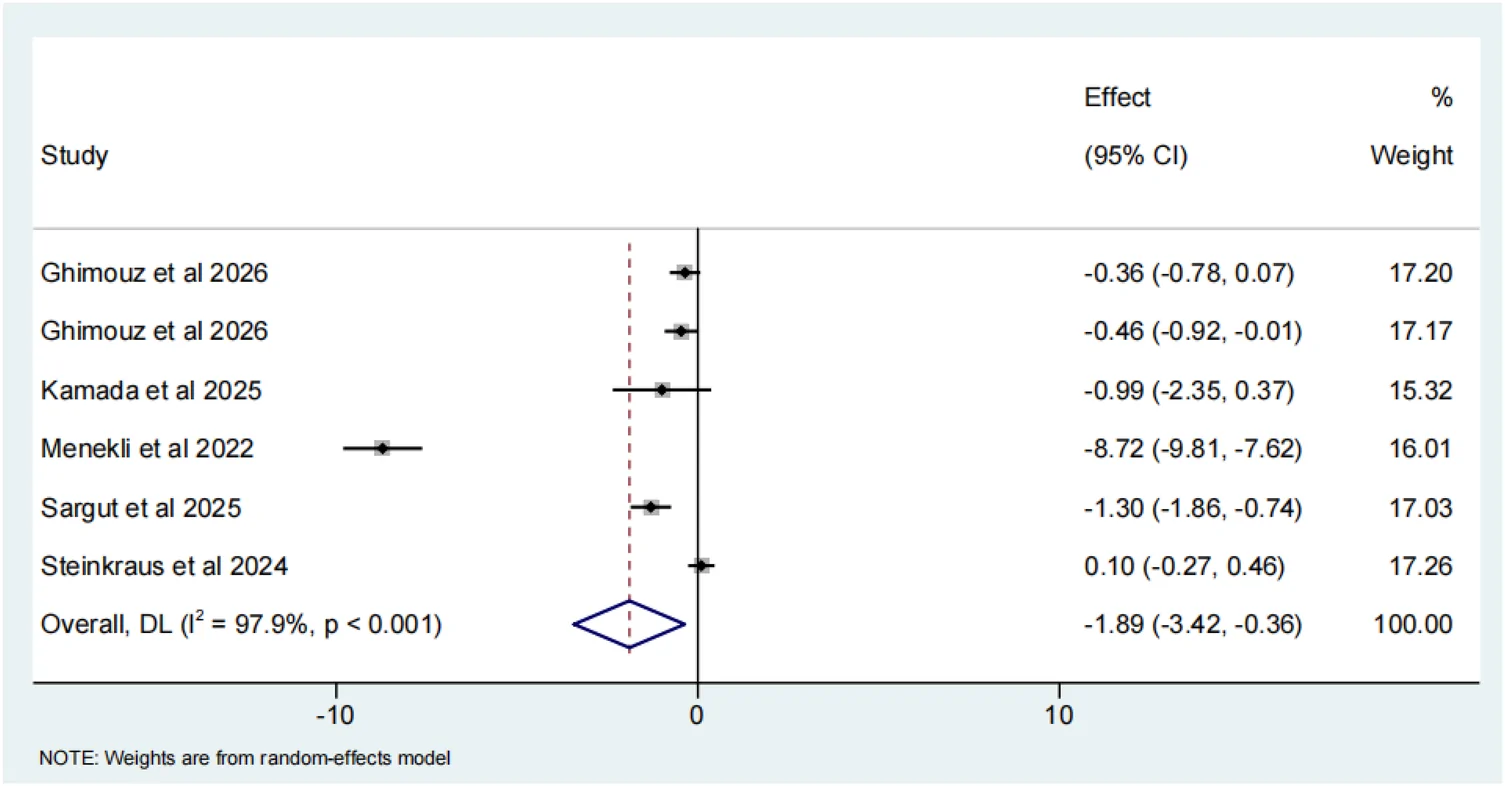
Forest plot showing the effect of VR intervention on anxiety scores of patients with TIVAP implantation. CI, confidence interval; DL, Random effects model; VR, virtual reality; TIVAP, totally implantable venous access port.

A total of 5 studies (15, 36–39) were included for anxiety scores. The heterogeneity test showed *I*^2^ = 97.90%, *P* < 0.001, prompting the use of a random-effects model. The meta-analysis demonstrated that VR intervention significantly reduced anxiety scores in patients undergoing TIVAP implantation [SMD = −1.89, 95% CI (−3.42, −0.36), *P* = 0.015] (Figure 4).

### Subgroup analysis

For pain scores, studies with a sample size of <100 showed a significant pain-reducing effect of VR intervention, whereas those with a sample size of ≥100 did not. When stratified by control group type, VR intervention did not significantly reduce pain scores regardless of whether the control group received standard care or music intervention. For anxiety scores, VR intervention significantly alleviated anxiety in both the <100 and ≥100 sample-size subgroups. When stratified by control group type, VR intervention showed a significant anxiety-reducing effect when the control group received music intervention, but did not reach statistical significance in the standard-care control group. The results of the subgroup analyses for each outcome are shown in Table 2.

**Table 2:** Subgroup analysis summary results.

Subgroup analysis	Heterogeneity		Meta-analysis	
	P	I^2^	SMD (95%CI)	P
Pain score				
Sample size				
<100	0.561	0.00%	**−1.07** (**−1.58, −0.56)**	**< 0.00001**
>100	< 0.00001	97.70%	−0.67 (−2.07, 0.72)	0.345
Type of intervention in the control group				
Standard care	< 0.00001	97.50%	−1.06 (−2.69, 0.57)	0.202
Music intervention	0.002	90.10%	−0.44 (−1.55, 0.68)	0.444
Anxiety score				
Sample size				
<100	0.681	0.00%	**−1.26** (**−1.78, −0.74)**	**< 0.00001**
>100	< 0.00001	98.70%	**−2.27** (**−4.39, −0.15)**	**0**.**036**
Type of intervention in the control group				
Standard care	< 0.00001	98.70%	−2.46 (−5.09, 0.18)	0.068
Music intervention	0.023	80.70%	**−0.87** (**−1.69, −0.05)**	**0**.**039**

The bold font indicates that the differences are statistically significant.

### Sensitivity analysis

**Figure 5:**
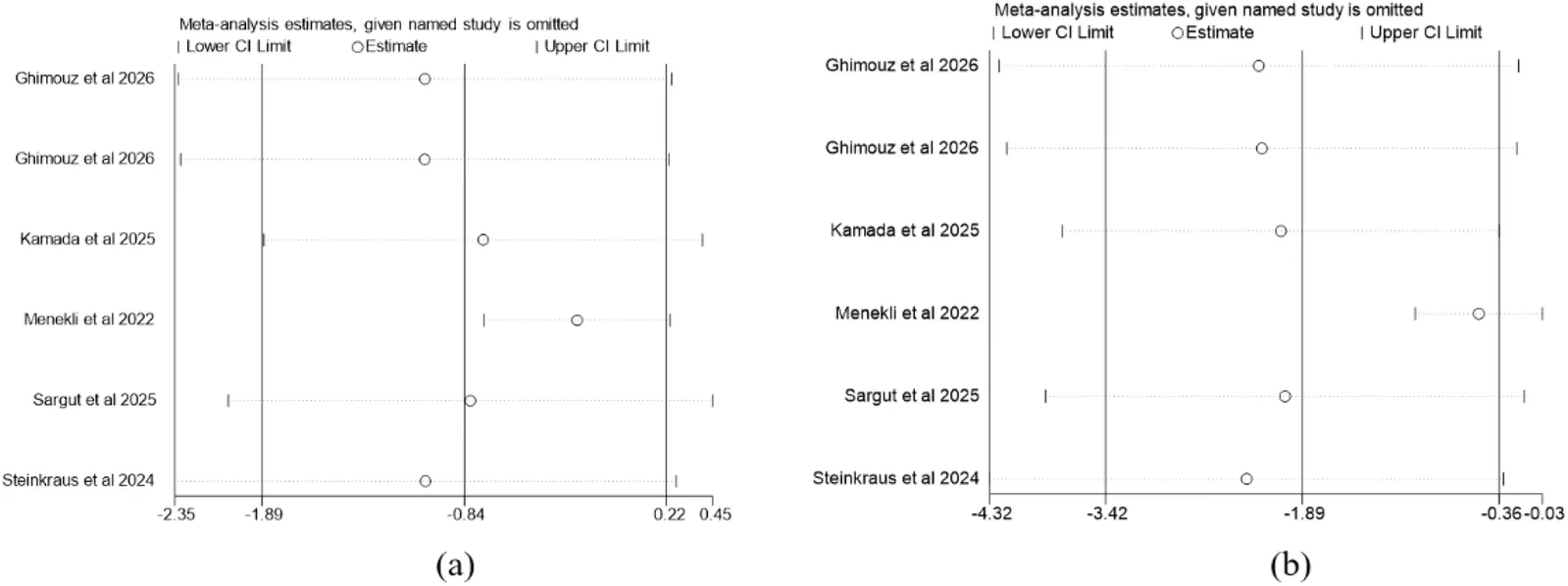
Sensitivity analysis results for the included studies. **(a)** pain scores; **(b)** anxiety scores. CI, confidence interval.

Sensitivity analysis using sequential exclusion of individual studies indicated that the pooled results were robust (Figure 5).

### Publication bias

**Figure 6:**
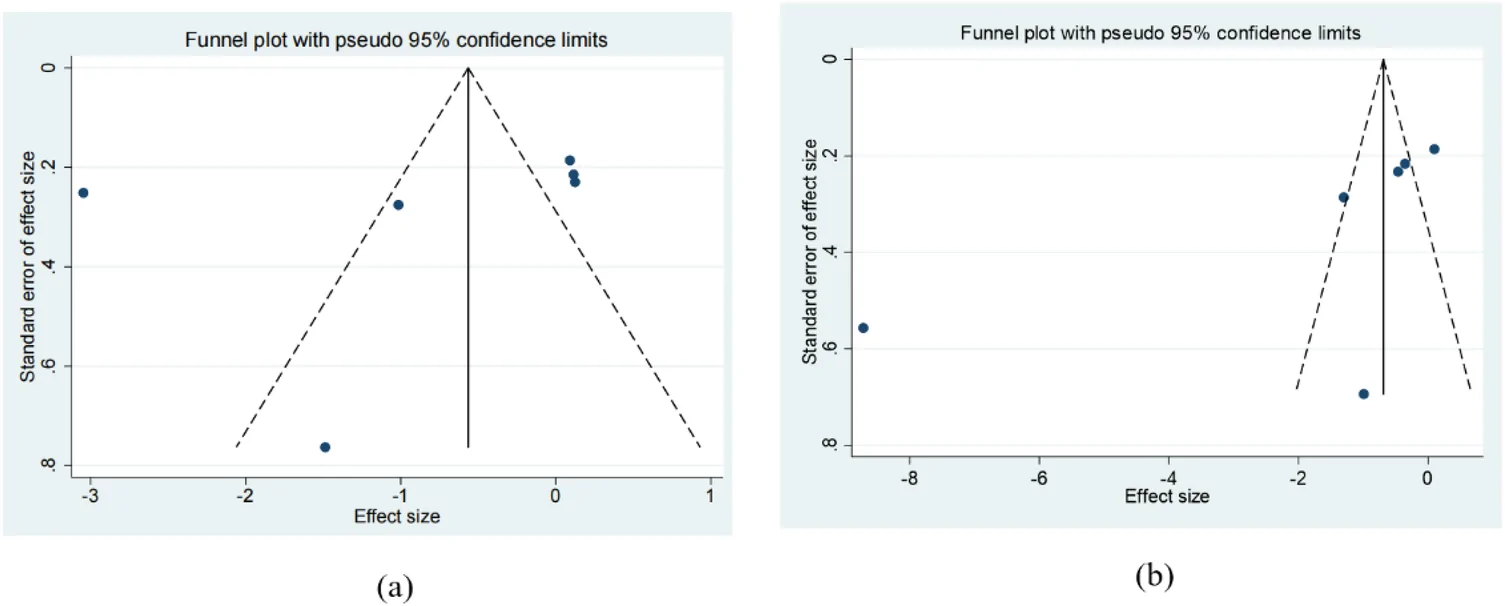
Publication bias analysis. **(a)** pain scores; **(b)** anxiety scores.

The scatter points on both sides of the funnel plot were not symmetrically distributed, which may suggest potential publication bias (Figure 6). However, given the limited number of included studies, this visual asymmetry should be interpreted with caution and cannot be taken as definitive evidence of publication bias.

### Evaluation of evidence quality

In the current meta-analysis, the certainty of evidence for pain scores was rated as very low, and that for anxiety scores as low (Table 3). The main factors leading to downgrading for both outcomes were the risk of bias (inability to blind participants and intervention personnel) and the very high heterogeneity across studies. These ratings indicate that, although the pooled effect for anxiety reached statistical significance, the overall confidence in the effect estimate remains low. Future high-quality, large-sample, well-designed RCTs with standardised VR intervention protocols are urgently needed to provide a more robust evidence base.

**Table 3:** Grading of recommendations assessment, development and evaluation systematic evaluation of evidence quality.

Outcomes	Quality assessment					SMD (95% CI)	Certainty of the evidence
	Risk of bias	Inconsistency	Indirectness	Imprecision	Publication bias		
Pain score	Serious^a^	Serious^b^	Not serious	Serious^c^	Not serious	SMD = −0.84, 95% CI (−1.89, 0.22)	Very low:⊕◯◯◯
Anxiety score	Serious^a^	Serious^b^	Not serious	Not serious	Not serious	SMD = −1.89, 95% CI (−3.42, −0.36)	Low:⊕⊕◯◯

a All the studies have a high risk of bias due to the inability to implement participant blinding. Moreover, the allocation and assessment blinding information was insufficient in most of the studies.

b There is extremely high heterogeneity among the various studies.

c The combined effect was not statistically significant, with a wide 95% confidence interval that crossed the null line.

## Discussion

This study conducted a systematic meta-analysis to evaluate the effects of VR intervention on anxiety and pain in patients undergoing TIVAP implantation. The results showed that VR intervention significantly improved anxiety scores, whereas no significant difference was observed for pain scores. This finding suggests that the effect of VR intervention in this population may be symptom-specific, with a stronger impact on emotional experiences (anxiety) than on physical sensations (pain)—a pattern worthy of further investigation.

Regarding the anxiety outcome, the VR intervention group demonstrated significantly lower anxiety levels than the control group, consistent with previous studies applying VR intervention to alleviate anxiety during procedures such as breast biopsy and wound dressing (13, 40, 41). This result is also in line with the majority of prior meta-analyses (42–46), which have consistently confirmed the anxiolytic effect of VR intervention across various invasive procedures—including percutaneous cardiovascular interventions, coronary angiography, cardiac surgery, puncture biopsy, and otolaryngology procedures (42–46). This suggests that the anxiety-relieving effect of VR intervention may be robust across different clinical scenarios. The immersive nature of VR intervention can effectively divert patients' attention away from concerns about puncture, device implantation, and potential complications, fostering a relaxed mental state and thereby reducing state anxiety. Subgroup analyses further revealed that VR intervention reduced anxiety regardless of sample size, and the effect was more pronounced when the control group received music intervention. This implies that the psychological relaxation effect of VR intervention may surpass that of simple auditory distraction, with its multi-sensory integrated immersive experience serving as a key advantage. During TIVAP implantation under local anesthesia, patients remain awake and are exposed to auditory and visual stimuli in the operating room, which may exacerbate anxiety. The highly immersive and distracting nature of VR intervention may help alleviate this distress, thereby improving the overall patient experience. Anxiety reduction may translate into higher patient satisfaction and more positive perceptions of the treatment process. Patients who feel calmer during the procedure may report greater satisfaction with nursing care—an increasingly important metric in value-based healthcare. For cancer patients undergoing TIVAP implantation, who often face repeated medical procedures and cumulative psychological burden, even transient relief of perioperative distress may contribute to improved psychological well-being. Previous studies have shown that VR intervention can reduce stress levels and help maintain more stable hemodynamic status during procedures performed under regional anesthesia (47). However, statistical significance should not be equated with clinical effectiveness, especially given the low strength of evidence in this study; over-reliance on P-values may mislead clinical decision-making. Although current evidence is insufficient to strongly recommend VR intervention during TIVAP implantation, if future high-quality studies confirm its anxiety-reducing effect, it could significantly improve patient experience, procedural tolerance, and perioperative care, particularly for those with high preoperative anxiety levels.

For the pain outcome, however, no statistically significant difference was found between the VR intervention and control groups. This result is not entirely consistent with previous studies suggesting the effectiveness of VR intervention in acute pain management (36, 48, 49). Our negative pain findings align with meta-analyses of percutaneous cardiovascular interventions and cystoscopy (42, 50), suggesting that VR intervention may have limited analgesic effects in certain invasive procedures under local anesthesia. Conversely, meta-analyses of coronary angiography, puncture biopsy, and otolaryngology procedures reported significant analgesic effects of VR intervention (43, 45, 46). The pain results of this study should be interpreted with caution and should not be construed as evidence that VR intervention is ineffective for pain management in TIVAP patients. The timing of pain assessment varied considerably across the included studies, ranging from intra-operative evaluation (15), immediate post-operative assessment (36, 39), to delayed assessment at 4 h post-surgery (37). This may have influenced the heterogeneity of effect estimates. However, because the number of included studies was too small to conduct a formal subgroup analysis by assessment time point, this explanation remains speculative. Second, the VR intervention content in the included studies was predominantly passive relaxation and nature scenes; only one study incorporated hypnotic guidance elements, and none used interactive game-based distraction. Emerging evidence suggests that interactive VR scenarios may produce stronger analgesic effects than passive relaxation scenes (51), and content specifically designed for pain management—such as active distraction and gamified elements—may be more effective than generic relaxation content (52). However, given the predominance of passive content among the included studies, a formal content-stratified analysis was not feasible, so this interpretation remains preliminary. In summary, although the observed null effect for pain does not support a definitive conclusion against VR's analgesic efficacy in TIVAP implantation, the above factors—heterogeneity in assessment timing and the predominance of passive VR intervention content—provide reasonable but unconfirmed explanations. These interpretations are intended to generate hypotheses for future research rather than to offer definitive explanations for the observed results.

The subgroup analyses further support the above inferences and reveal several important findings. In terms of pain scores, studies with a sample size of less than 100 showed that VR intervention significantly reduced pain, whereas studies with a sample size of more than 100 did not, suggesting that small-sample studies may be subject to publication bias or effect overestimation, and that larger studies tend to yield more conservative estimates. However, because formal statistical assessment of publication bias was not feasible, this difference may also be influenced by the small number of included studies and substantial heterogeneity in VR intervention protocols across studies. Therefore, the effect differences between subgroups should not be attributed to sample size or publication bias; the exact reasons require verification in future larger, methodologically harmonized studies. Additionally, when stratified by the type of control group, neither the standard care nor the music intervention group reached statistical significance, indicating that VR's analgesic effect in TIVAP implantation was neither superior to conventional care nor to simple music distraction. This may suggest that under adequate local anesthesia, any distraction method has limited additional benefit for pain that is already well controlled. In terms of anxiety scores, studies with ≥100 participants also showed a significant effect of VR intervention, albeit with a smaller effect size than small-sample studies, but the direction was consistent, suggesting that the evidence for anxiety improvement is relatively robust. However, the anxiety effect of VR intervention did not reach significance in the standard care control group, whereas it was significant in the music intervention control group. This contradictory phenomenon may be influenced by the small number of included studies, differing baseline anxiety levels, and variations in nursing measures within the standard care group, and should therefore be interpreted with caution. All subgroup analyses should be viewed as exploratory hypothesis-generating analyses rather than confirmatory conclusions.

The pooled effect sizes of this study should be interpreted within the dual context of extremely high heterogeneity and low GRADE evidence quality. Together, these factors undermine the internal validity and clinical generalizability of the results. The very high I² value suggests that the effect differences across studies are likely driven by systematic differences in device type, intervention content, duration, assessment tools, operator experience, and disease characteristics, rather than by sampling error alone. Although the random-effects model can produce a point estimate of the “average effect,” this average is highly dependent on the specific combination of included studies and may not represent the true effect in any given clinical scenario; it may even mask differences in effect direction across subgroups. Although the pooled anxiety effect was negative, individual studies had very wide confidence intervals, making the average difficult to apply to individualized decision-making. The included studies used different devices, such as PICO G2 4K and smartphone-compatible goggles, with variations in resolution and field of view that may directly affect immersion. Intervention content ranged from “underwater whale watching” to “space roaming,” with different levels of active guidance and relaxation strategies, potentially influencing psychological engagement and distraction effectiveness. Some studies initiated VR intervention only 1 min before surgery, while others continued throughout the entire procedure or even up to 4 h post-operatively; baseline pain and anxiety levels vary at different time points, and thus intervention effects naturally differ. Some studies used headphones for auditory isolation, while others used built-in speakers to maintain clinician-patient communication; the degree of auditory isolation affects immersion and intra-operative cooperation, thereby interfering with effect assessment. Pain was assessed using NRS, VAS, and McGill questionnaires, while anxiety was assessed using STAI-6, SAI, NRS, and other tools; these instruments differ in sensitivity and dimensionality. Assessment time points (intra-operative, immediately post-operative, and several hours post-operative) were not uniform across studies, potentially capturing responses at different pathophysiological phases. Tumor types (breast, colorectal, lung, etc.) and patient age/sex composition may influence baseline anxiety levels and VR acceptance, thereby altering effect sizes. Differences in institutional TIVAP implantation proficiency, local anesthesia practices, and preoperative education content may change patients' reference points for pain and anxiety perception, thus confounding net effect estimates. The GRADE evidence quality was rated as “very low” or “low,” indicating low confidence in the effect estimates and that the true effects may differ substantially from the current point estimates. The GRADE system identifies “risk of bias” as the primary downgrading factor. Inadequate allocation concealment may introduce selection bias, whereby investigators may consciously or unconsciously assign patients with better prognoses or greater acceptance to the VR intervention group, thereby exaggerating the intervention effect. Even if random sequence generation is appropriate, inadequate allocation concealment may still permit this potential bias. Due to the nature of VR interventions, none of the studies could blind participants or personnel. Patients in the VR intervention group were aware that they were receiving a “new technological intervention,” which may have created a placebo effect through expectation, leading to more favorable self-reported anxiety and pain scores. For pain (statistically non-significant), very low evidence means that we can neither rule out a potential analgesic effect nor confirm its absence; the correct interpretation is “insufficient evidence to reach a definitive conclusion” rather than “VR intervention is ineffective.” For anxiety (statistically significant), low evidence suggests that the significance may partially result from overestimation in small-sample studies, performance bias due to inability to blind, and methodological variability, with a risk of false-positive findings. Thus, statistical significance should not be equated with clinical effectiveness, especially when evidence strength is insufficient; over-reliance on *P*-values may mislead decisions. The combination of very high heterogeneity and low evidence quality suggests that the results of this meta-analysis are more suitable as a basis for exploratory hypothesis generation than for definitive clinical recommendations. Its core value lies in clearly revealing the current disagreements and methodological shortcomings in the existing literature, thereby pointing the way toward future standardized, large-sample, multicenter RCTs. Subsequent studies should standardize VR intervention protocols (device, content, timing, duration), adopt validated and uniform assessment tools, implement allocation concealment and assessor blinding, so as to reduce heterogeneity and upgrade evidence quality, thereby providing more reliable evidence for clinical practice.

The initial procurement cost of VR headsets and accompanying software is relatively high, and the development of high-quality VR intervention content requires sustained investment by professional teams, potentially increasing departmental budget pressures. Formal health economic evaluations specifically for TIVAP implantation are currently lacking and should be a priority for future research. The use of VR equipment requires specialized training for healthcare providers, including device fitting, parameter setting, content selection, and emergency troubleshooting, all of which add extra workload. In busy oncology day-surgery centers, routine implementation necessitates dedicated personnel; otherwise, surgical turnover efficiency may be compromised. We recommend that future studies develop standardized operating procedures to reduce training costs and operational variability. VR intervention may interfere with intra-operative clinician-patient communication (e.g., when headphones are used); some studies used speakers instead to maintain communication but at the cost of immersion. A balance must be struck between “distraction” and “medical safety.” Moreover, the preparation, disinfection, and startup of VR equipment require additional time, which may extend the procedure turnaround; this can be mitigated through process optimization (e.g., pre-fitting in the preoperative preparation area). Reusable VR headsets come into direct contact with patients' facial skin, posing a risk of cross-infection. Strict cleaning and disinfection protocols must be established. This increases consumable costs and nursing workload and may reduce headset lifespan. Future alternatives, such as disposable covers or dedicated isolation masks, require further evaluation of their economic and feasibility aspects. Some patients may be unable to complete the full intervention due to VR intervention-related discomfort (e.g., dizziness, nausea, visual fatigue), especially elderly patients or those with a history of motion sickness. A pre-intervention tolerance screening process should be established before clinical roll-out, along with contingency plans during the intervention. In practice, patients should be fully informed of potential discomfort before the procedure and allowed to choose whether to receive VR intervention, thereby respecting patient autonomy and improving acceptability. Most current studies have been conducted under relatively ideal conditions. In real-world clinical settings, with high surgical volumes, rapid turnover, and constrained healthcare human resources, the management, disinfection, and operational guidance of VR equipment all demand additional time and personnel. Without dedicated device management support, embedding VR intervention into routine clinical work will face substantial practical challenges.

## Limitations

This study has several limitations. First, the number of included studies and the overall sample size were limited. Only 5 RCTs (totaling 452 participants) were included, with most being single-center studies and sample sizes varying considerably. Consequently, some subgroup analyses included only 2 studies, yielding insufficient statistical power for reliable conclusions. Moreover, with fewer than 10 included studies, we could not perform Egger's regression test to quantify publication bias; visual inspection of the funnel plot has low reliability and should be interpreted with caution. Second, high methodological heterogeneity limited the interpretability of the pooled effects. There were substantial differences across studies in VR device models, intervention content, timing, and duration, leading to poor comparability. Although statistically feasible, the pooled effect size may not represent the true effect of any specific protocol. Third, the outcome assessment tools were not uniform, increasing measurement heterogeneity. Pain was assessed using three tools (NRS, VAS, McGill), and anxiety using four (NRS, VAS, SAI, STAI-6). Although we used SMD for statistical conversion, the clinical equivalence among these instruments lacks empirical support, potentially introducing measurement error and undermining the clinical interpretability of the pooled effect. Fourth, bias control was insufficient, affecting internal validity. Due to the inherent characteristics of VR interventions, none of the studies could blind participants or personnel; most studies did not adequately report allocation concealment or assessor blinding, making it difficult to rule out selection and detection bias. Furthermore, as anxiety and pain are both patient-reported subjective outcomes, they are susceptible to expectation effects and psychological suggestion in the absence of blinding, potentially exaggerating the VR effect. Fifth, the evidence quality was low, and confidence in the effect estimates was limited. Based on the GRADE assessment, the evidence quality for pain was “very low” and for anxiety “low,” with the main downgrading factors being risk of bias and very high heterogeneity. This means that the true effects may substantially deviate from the current point estimates; statistical significance of the pooled results should not be equated with clinical effectiveness, and overinterpretation should be avoided.

## Conclusion

Based on current evidence, the analgesic effect of VR intervention in patients undergoing TIVAP implantation remains uncertain. The pooled estimate did not reach significance; this should not be interpreted as evidence of inefficacy, but rather as reflecting insufficient evidence to draw a definitive conclusion. Given the low quality of current evidence, the role of VR intervention in alleviating anxiety during TIVAP implantation should be viewed cautiously and cannot yet be recommended as a routine adjunct; future high-quality studies are needed for confirmation. Pending further evidence, clinicians should regard VR intervention as an unproven pain management tool, to be used only in research settings or within individualized clinical decision-making contexts, with patients fully informed of the current uncertainties. Future research should prioritize the following directions to enhance comparability, reproducibility, and clinical utility: First, standardize the timing of outcome assessment. Recommended time points include baseline (pre-operative), intra-operative or immediately post-operative, pre-discharge (approximately 2 h post-operatively), and short-term follow-up (e.g., 7–14 days). Second, harmonize VR intervention protocols to reduce content- and delivery-related heterogeneity: (a) clearly define immersion type (immersive vs. semi-immersive); (b) describe intervention content in sufficient detail (relaxation-oriented vs. active distraction vs. hypnotic guidance); (c) standardize VR exposure timing and duration (throughout the procedure vs. specific phases only); and (d) specify audio delivery mode (headphones vs. built-in speakers). Third, extend follow-up duration to evaluate the sustainability of VR effects. Perioperative anxiety and pain are typically acute, but long-term psychological outcomes—such as procedure-related fear, treatment adherence, and patient satisfaction with future procedures—remain under-assessed. Fourth, adopt uniform, validated outcome assessment tools. Future studies should prioritize well-validated instruments with established minimal clinically important differences to facilitate clinical interpretation of effect sizes. Finally, future RCTs should be designed with adequate statistical power, clear reporting of randomization and allocation concealment, and assessor blinding (where feasible) to address the methodological limitations identified in the current evidence base.

## Data Availability

The datasets presented in this study can be found in online repositories. The names of the repository/repositories and accession number(s) can be found in the article/Supplementary Material.
