# Supplementary material for: The effect of virtual reality on anxiety and pain in patients undergoing totally implantable venous access port: a systematic review and meta-analysis
Source: Front Med (Lausanne). 2026 Sep 8;13:1923678. doi: 10.3389/fmed.2026.1923678 (PMC13593795; doi:10.3389/fmed.2026.1923678)
Supplement: Supplementary file 1 [file Table1.docx]

| **Supplementary Materials Table S1. Search strategy** | |
| --- | --- |
| Pubmed | ("Virtual Reality"[Mesh] OR "virtual reality"[Title/Abstract] OR "VR"[Title/Abstract] OR "immersive virtual reality"[Title/Abstract] OR "head-mounted display"[Title/Abstract] OR "HMD"[Title/Abstract])AND("totally implantable venous access port"[Title/Abstract] OR "TIVAP"[Title/Abstract] OR "totally implantable venous access device"[Title/Abstract] OR "TIVAD"[Title/Abstract] OR "central venous port"[Title/Abstract] OR "port catheter"[Title/Abstract] OR "implantable venous access port"[Title/Abstract] OR "port-a-cath"[Title/Abstract] OR "venous port"[Title/Abstract] OR "implantable port"[Title/Abstract])AND("Anxiety"[Mesh] OR "anxiety"[Title/Abstract] OR "anxious"[Title/Abstract] OR "anxiousness"[Title/Abstract] OR "Pain"[Mesh] OR "pain"[Title/Abstract] OR "painful"[Title/Abstract])AND("Randomized Controlled Trial"[Publication Type] OR "randomized controlled trial"[Title/Abstract] OR "RCT"[Title/Abstract] OR "randomised"[Title/Abstract] OR "randomized"[Title/Abstract] OR "controlled trial"[Title/Abstract]) |
| Cochrane Library | #1 MeSH descriptor: [Virtual Reality] explode all trees #2 ("virtual reality" OR "VR" OR "immersive virtual reality" OR "head-mounted display" OR "HMD"):ti,ab,kw #3 #1 OR #2 #4 MeSH descriptor: [Central Venous Catheters] explode all trees #5 MeSH descriptor: [Catheterization, Central Venous] explode all trees #6 ("totally implantable venous access port" OR "TIVAP" OR "totally implantable venous access device" OR "TIVAD" OR "central venous port" OR "port catheter" OR "implantable venous access port" OR "port-a-cath" OR "venous port" OR "implantable port"):ti,ab,kw #7 #4 OR #5 OR #6 #8 MeSH descriptor: [Anxiety] explode all trees #9 ("anxiety" OR "anxious" OR "anxiousness"):ti,ab,kw #10 #8 OR #9 #11 MeSH descriptor: [Pain] explode all trees #12 ("pain" OR "painful"):ti,ab,kw #13 #11 OR #12 #14 ("randomized controlled trial" OR "randomised controlled trial" OR "RCT" OR "randomized" OR "randomised" OR "controlled trial"):ti,ab,kw #15 #3 AND #7 AND #10 AND #13 AND #14 |
| Embase | ('virtual reality'/exp OR 'virtual reality':ab,ti OR vr:ab,ti OR 'immersive virtual reality':ab,ti OR 'head-mounted display':ab,ti OR hmd:ab,ti) AND ('implantable venous access device'/exp OR 'central venous catheter'/exp OR 'totally implantable venous access port':ab,ti OR tivap:ab,ti OR 'totally implantable venous access device':ab,ti OR tivad:ab,ti OR 'central venous port':ab,ti OR 'port catheter':ab,ti OR 'port-a-cath':ab,ti OR 'venous port':ab,ti OR 'implantable port':ab,ti) AND ('anxiety'/exp OR anxiety:ab,ti OR anxious:ab,ti) AND ('pain'/exp OR pain:ab,ti OR painful:ab,ti) AND ('randomized controlled trial'/exp OR 'randomized controlled trial':ab,ti OR 'randomised controlled trial':ab,ti OR rct:ab,ti OR randomized:ab,ti OR randomised:ab,ti OR 'controlled trial':ab,ti) |
| Web of Science | TS=("virtual reality" OR VR OR "immersive virtual reality" OR "head-mounted display" OR HMD) AND TS=("totally implantable venous access port" OR TIVAP OR "totally implantable venous access device" OR TIVAD OR "central venous port" OR "port catheter" OR "implantable venous access port" OR "port-a-cath" OR "venous port" OR "implantable port") AND TS=(anxiety OR anxious OR anxiousness) AND TS=(pain OR painful) AND TS=("randomized controlled trial" OR "randomised controlled trial" OR RCT OR randomized OR randomised OR "controlled trial") |
